# Cognitive functioning in everyday life: The development of a questionnaire on instrumental activities of daily living in multiple sclerosis

**DOI:** 10.1177/20552173211038027

**Published:** 2021-08-03

**Authors:** Maureen van Dam, Sietske AM Sikkes, Emma Rammeloo, Evy Reinders, Julia R Jelgerhuis, Jeroen JG Geurts, Bernhard MJ Uitdehaag, Hanneke E Hulst

**Affiliations:** Department of Anatomy and Neurosciences, MS Center Amsterdam, Amsterdam Neuroscience, Amsterdam UMC, Vrije Universiteit Amsterdam, Amsterdam, The Netherlands; Department of Neurology, Alzheimer Center Amsterdam, Amsterdam Neuroscience, Amsterdam UMC, Vrije Universiteit Amsterdam, Amsterdam, The Netherlands; Department of Epidemiology and Biostatistics, Amsterdam Neuroscience, Amsterdam UMC, Vrije Universiteit Amsterdam, Amsterdam, The Netherlands; Department of Anatomy and Neurosciences, MS Center Amsterdam, Amsterdam Neuroscience, Amsterdam UMC, Vrije Universiteit Amsterdam, Amsterdam, The Netherlands; Department of Epidemiology and Biostatistics, Amsterdam Neuroscience, Amsterdam UMC, Vrije Universiteit Amsterdam, Amsterdam, The Netherlands; Department of Neurology, MS Center Amsterdam, Amsterdam Neuroscience, Amsterdam UMC, Vrije Universiteit Amsterdam, Amsterdam, The Netherlands; 1 Department of Anatomy and Neurosciences, MS Center Amsterdam, Amsterdam Neuroscience, Amsterdam UMC, Vrije Universiteit Amsterdam, Amsterdam, The Netherlands

**Keywords:** Multiple sclerosis, cognition, instrumental activities of daily living, Amsterdam IADL Questionnaire, MS-IADL-Q

## Abstract

Neuropsychological test scores in people with MS (PwMS) do not fully reflect cognitive functioning in daily life. Therefore, we developed a questionnaire based on instrumental activities of daily living (IADL), using the Amsterdam IADL-Q^©^ for Alzheimer’s disease as starting point. Forty-eight items were evaluated on relevance and clarity by (inter)national experts (n = 30), PwMS (n = 61) and proxies (n = 30). Consequently, four items were omitted, two items were merged and seven items were added. Fifty items were included in the IADL questionnaire specific to cognitive functioning in MS (the MS-IADL-Q). Future studies are warranted to assess the psychometric properties of the MS-IADL-Q.

## Introduction

Up to 70% of the people with Multiple Sclerosis (PwMS) experience cognitive impairment,^1^ which substantially impacts daily functioning, work participation and quality of life.^2^

Neuropsychological examination is currently the “gold standard” to assess cognitive functioning, although its ecological validity is being questioned.^1^ Cognitive tasks in daily life often have to be performed in an environment with distractors and are consequently more demanding than cognitive tests in a clinical setting.^1^ Alternatively, self-report questionnaires on cognitive performance (e.g. Multiple Sclerosis Neuropsychological Questionnaire (MSNQ)) are only weakly correlated with neuropsychological test scores and seem to reflect fatigue and mood instead.^3^

In Alzheimer’s disease (AD) and neuro-oncology, questionnaires measuring “instrumental activities of daily living” (IADL) bridge the gap between functioning in daily life and neuropsychological test scores.^4–6^ IADLs are defined as complex tasks that require multiple cognitive processes to be active,^4^ and are known to be susceptible to early signs of cognitive decline.^7^ In AD, this questionnaire has been validated and correlated with neuropsychological test results,^6^ consequently enabling the detection of small changes in daily cognitive functioning (pre-disease status).^8^ Therefore, an IADL questionnaire for PwMS (the MS-IADL-Q) is expected to link cognitive functioning in everyday life to clinical measures of cognition enabling timely detection of cognitive decline.

## Methods

### Step 1: Item-selection for the MS-IADL-Q

**Figure 1.**
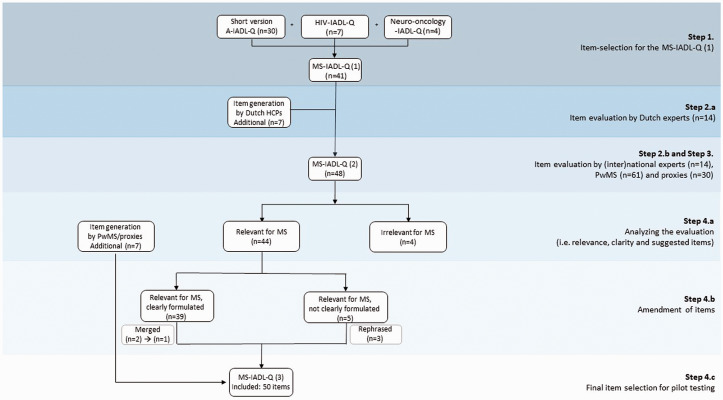
Flowchart reflecting the steps taken to develop and evaluate the MS Instrumental Activities of Daily Living Questionnaire (the MS-IADL-Q). PwMS: people with MS.

The first step included the composition of the MS-IADL-Q based on the short version of the well-validated Amsterdam IADL Questionnaire (A-IADL-Q) developed for people with AD^9^ and the recently developed IADL questionnaires for patients with brain tumors^5^ and patients with HIV (in preparation; Figure 1, online Appendix A). Both a Dutch and English version of the questionnaire were developed.

### Step 2: Item evaluation by experts

To ensure content validity, the items for the questionnaire were evaluated by national (step 2a) and international experts (step 2 b), i.e., neurologists, neuropsychologists, neuroscientists, nurses, rehabilitation physicians and occupational therapists. The relevance of the items was evaluated on a visual analogue scale ranging from 0 (“not relevant at all”) to 100 (“very relevant”). Additionally, the clarity of the item description was evaluated (open ended question) and potential missing items could be added (online Appendix B).

### Step 3: Evaluation by PwMS and proxies

The version of the MS-IADL-Q adjusted by the experts (online Appendix D) was then evaluated by PwMS and proxies as described in step 2 (online Appendix C). Medical Ethical approval was obtained from the VU University Medical Center.

### Step 4: Final item selection

The feedback from step 2 and step 3 was merged. Items with a mean rating of ≥75 were classified as “highly relevant”, items with a score of ≥60 and <75 were classified as “moderately relevant”, and items with a score of <60 were classified “little relevance”.^9^ If items received a score of <60 (i.e., “irrelevant”) by all groups, exclusion from the questionnaire was justified.^9^ All items with a “moderate to high” rating (>60) and no more than six “unclear” ratings were included in the final selection. Unclear items were evaluated and subsequently omitted or rephrased. Suggestions were incorporated in the questionnaire if the suggestion was mentioned at least three times by an independent rater.

## Results

### Step 1: Item-selection for the MS-IADL-Q

Thirty items of the A-IADL-Q, four items of the neuro-oncology list and seven items of the HIV list were included in the first version of the MS-IADL-Q (41 items) covering the following IADL: household, appliances, administration, work, devices, leisure, transport, and “other” activities (online Appendix A).

### Step 2: Item evaluation by experts

Seven items were added to the list: “keeping appointments”, “focusing attention while performing tasks at work”, “dealing with distractions at work”, three items related to smartphone-use, and “other participation in traffic”. The new MS-IADL-Q (48 items) was evaluated by the international experts (n = 15) and one more national expert (online Appendix D). No differences in expert ratings were found between national and international experts (online Appendix B).

### Step 3: Evaluation by PwMS and proxies

Sixty-one PwMS (67% female, mean age = 49.0 ± 10.2SD, relapsing remitting MS (60%), progressive MS (28%), other (12%)) and 30 proxies (57% female, mean age = 51.4 ± 12.8SD) evaluated the 48 items of the MS-IADL-Q (Figure 1).

### Step 4: Final item selection

*Relevance* | The experts considered 35/48 items (73%) relevant to PwMS, whereas PwMS and proxies considered 46/48 items (96%) and 45/48 items (94%) as relevant, respectively (Table 1; online Appendix E). Four items (i.e., “playing card and board games”, “playing computer games”, “paying with cash” and “making minor repairs to the house”) received a score <60 by all groups and were therefore omitted from the questionnaire.

**Table 1.** Evaluation of the MS-IADL-Q (version 2) by experts (national and international), People with MS (PwMS) and their proxies (ordered from highest average relevance to lowest average relevance). The average relevance per group is displayed, together with the mean. For relevance, scores range from 0 (“not relevant at all”) to 100 (“very relevant”). For clarity of the item, frequency per group is displayed, together with the sum.

		Relevance(0–100)				Clarity			
Category	Activity	Experts (n = 30)	PwMS (n = 61)	Proxies (n = 30)	Mean	Experts (n = 30)	PwMS (n = 61)	Proxies (n = 30)	Sum
4	3. Focusing attention while performing tasks at work	91.10	88.03	76.87	85.33	1	2	1	4
3	5. Keeping appointments	78.80	88.80	82.73	83.45	0	3	2	5
5	1. Using a computer	80.80	86.77	82.60	83.39	1	1	3	5
3	4. Making appointments	79.70	86.67	80.67	82.35	1	3	2	6
5	6. Using a mobile phone or smartphone	79.70	83.97	82.00	81.89	0	0	1	1
8	2. Being responsible for his/her own medication	75.80	88.34	81.07	81.74	0	1	0	1
8	5. Learning new things (such as a course, computer program, or appliance)	84.50	83.00	75.70	81.07	1	0	1	2
4	1. Working	89.20	81.51	70.80	80.50	1	2	4	7
8	4. Having a conversation with multiple people at the same time	82.60	81.03	77.57	80.40	2	0	1	3
3	6. Using a PIN-code	69.30	85.90	85.90	80.37	0	0	0	0
7	1. Driving a car	78.90	83.08	78.67	80.22	0	1	3	4
3	1. Paying bills	75.20	83.48	81.23	79.97	0	1	1	2
6	4. Reading a book or newspaper	78.40	82.26	78.90	79.85	0	1	0	1
4	4. Dealing with distractions at work	86.70	83.39	69.13	79.74	1	3	1	5
3	2. Managing the household budget	78.00	82.97	77.20	79.39	0	1	1	2
7	4. Other participation in traffic (for instance by foot, bike, or scoot mobile)	65.40	89.48	82.40	79.09	2	1	3	6
3	3. Using electronic banking	69.50	84.16	80.37	78.01	0	0	0	0
5	2. E-mailing	68.30	84.61	80.73	77.88	0	0	3	3
3	9. Filling in forms	69.50	84.54	78.43	77.49	2	1	1	4
1	4. Cooking	74.20	80.23	77.33	77.25	0	1	2	3
6	3. Following a TV program or movie	72.60	78.25	80.37	77.07	0	1	0	1
8	3. Doing multiple things at the same time (multitasking)	80.80	78.15	71.37	76.77	1	0	1	2
1	1. Carrying out household duties	74.50	79.44	75.80	76.58	4	3	4	11
5	7. Making phone calls with a mobile phone or smartphone	67.30	82.87	78.73	76.30	0	0	0	0
1	3. Buying the correct groceries	70.20	81.36	76.23	75.93	0	0	3	3
1	2. Grocery shopping independently	72.50	80.13	75.13	75.92	0	1	2	3
5	4. Operating devices	68.20	80.64	76.17	75.00	8	5	7	20
4	2. Finishing work on time	83.10	77.43	63.07	74.53	3	1	5	9
6	5. Organizing/initiating social activities	76.50	76.51	69.97	74.32	0	1	1	2
7	3. Using public transport	73.50	77.67	70.80	73.99	0	2	3	5
3	10. Making online purchases (on any device)	66.70	83.48	70.30	73.49	0	2	1	3
7	2. Using a navigation system	66.80	78.85	73.57	73.07	0	1	1	2
8	1. Using keys	58.80	79.02	77.13	71.65	2	0	0	2
8	6. Writing in any format	68.10	73.16	71.20	70.82	4	0	4	8
5	5. Operating the television remote control	58.20	76.67	75.83	70.24	0	0	1	1
2	2. Operating the microwave oven	59.50	72.95	77.13	69.86	0	0	3	3
2	1. Operating domestic appliances	59.20	76.18	73.67	69.68	1	1	3	5
1	5. Preparing cold meals	58.70	71.18	69.70	66.53	3	0	2	5
2	3. Operating the coffee maker	58.30	67.98	73.20	66.49	1	0	3	4
2	4. Operating the washing machine	60.00	72.07	63.43	65.17	1	4	8	13
3	7. Obtaining money from an ATM	66.60	67.46	60.40	64.82	0	0	0	0
5	9. Sending out e-mails on a smartphone	54.90	68.82	66.93	63.55	0	1	3	4
5	8. Using social media on a smartphone	53.40	69.02	67.27	63.23	0	1	2	3
5	3. Printing documents	55.40	62.23	65.57	61.07	0	0	1	1
3	8. Paying with cash	59.20	63.92	56.47	59.86	0	0	0	0
6	1. Playing card and board games	57.60	59.57	59.83	59.00	0	1	1	2
1	6. Making minor repairs to the house	44.70	64.67	62.07	57.15	0	1	2	3
6	2. Playing computer games	55.20	51.80	56.57	54.52	0	1	1	2

*Clarity* | Thirty-nine relevant items were clearly formulated (Table 1; online Appendix F). Five items were unclear, of which three items were rephrased and two items were improved by editing the format of the questionnaire. Due to the overlap, two items (“E-mailing” and “Sending out e-mails on a smartphone”) were merged into one item (“sending out e-mails”).

*Suggested items* | Seven novel items were suggested by at least three participants (Figure 1, online Appendix F). The final version of the MS-IADL-Q consists of 50 items (online Appendix G).

## Discussion

A questionnaire for PwMS was developed to investigate cognitive performance in daily life using IADL. Relevant items were selected by (inter)national experts, PwMS and their proxies, resulting in 50 items for the final MS-IADL-Q. National and international experts did not differ in their ratings, suggesting that the MS-IADL-Q can be used in an international setting.

During the item-selection, concerns were expressed that physical problems, rather than cognitive problems, would interfere with IADL. Therefore, in the final version of the MS-IADL-Q, a question will be added to differentiate between physical and cognitive problems.

The A-IADL-Q was used as a starting point for questionnaire development because of its previously confirmed psychometric properties,^4,6^ such as the ability to detect treatment effects and small cognitive changes between groups and over time.^9,10^ We expect that the MS-IADL-Q has similar psychometric properties in PwMS and allows for remote and low-key use. Next, the MS-IADL-Q needs to be validated in different MS-subtypes, disease durations and over time. Ideally, this will be done in an international set-up.

Interested in participating in collecting data on MS-IADL-Q in your own country? Please let us know (m.vandam2@amsterdamumc.nl) and join our team.

## Conflict of Interests

The author(s) declared the following potential conflicts of interest with respect to the research, authorship, and/or publication of this article:

M.v.D. is supported by a research grant from Celgene.

S.A.M.S. reports license fees for use of the Amsterdam IADL Questionnaire (Green Valley, VtV Therapeutics, Alzheon, Vivoryon, Roche), Consultancy fees from Boehringer and Toyama: all funds and license fees were paid to the institution. Academic funding through Health Holland (OTAPA, LSHM19051; DEFEAT-AD, LSHM20084).

E.Ra, E.Re and J.R.J. report no conflicts of interest.

J.J.G. is an editor of Multiple Sclerosis Journal. He serves on the editorial boards of Neurology and Frontiers in Neurology and is president of the Netherlands organization for health research and innovation. He has served as a consultant for or received research support from Biogen, Celgene, Genzyme, MedDay, Merck, Novartis and Teva.

B.M.J.U. reports personal fees for consultancies from Biogen Idec, Genzyme, Merck Serono, Novartis, Roche, and Teva, outside the submitted work.

H.E.H. serves on the editorial board of Multiple Sclerosis Journal and has received compensation for consulting services or speaker honoraria from Celgene, MedDay, Sanofi-Genzyme, Merck B.V. and Biogen.

## Supplemental Material

sj-pdf-1-mso-10.1177_20552173211038027 - Supplemental material for Cognitive functioning in everyday life: The development of a questionnaire on instrumental activities of daily living in multiple sclerosis Click here for additional data file. Supplemental material, sj-pdf-1-mso-10.1177_20552173211038027 for Cognitive functioning in everyday life: The development of a questionnaire on instrumental activities of daily living in multiple sclerosis by Maureen van Dam Sietske AM Sikkes Hanneke E Hulst in Multiple Sclerosis Journal – Experimental, Translational and Clinical
