# Supplementary material for: Cognitive functioning in everyday life: The development of a questionnaire on instrumental activities of daily living in multiple sclerosis
Source: Mult Scler J Exp Transl Clin. 2021 Aug 3;7(3):20552173211038027. doi: 10.1177/20552173211038027 (PMC8365017; doi:10.1177/20552173211038027)
Supplement: sj-pdf-1-mso-10.1177_20552173211038027 - Supplemental material for Cognitive functioning in everyday life: The development of a questionnaire on instrumental activities of daily living in multiple sclerosis [file sj-pdf-1-mso-10.1177_20552173211038027.pdf]

Supplementary materials

Appendix A. First version of the MS-IADL-Q (after step 1; 41 items)

From the Alzheimer's questionnaire (30 items)[9]:

1. Household duties

(1.1) Carrying out household duties

(1.2) Grocery shopping independently

(1.3) Buying the correct groceries

(1.4) Cooking

(1.5) Preparing cold meals

(1.6) Making minor repairs to the house

2. Using household appliances

(2.1) Operating domestic appliances

(2.2) Operating the microwave oven

(2.3) Operating the coffee maker

(2.4) Operating the washing machine

3. Administration

(3.1) Paying bills

(3.2) Managing the household budget

(3.3) Using electronic banking

(3.4) Making appointments

(3.5) Using a PIN code

(3.6) Obtaining money from an ATM

(3.7) Paying with cash

(3.8) Filling in forms

4. Working

(4.1) Working

5. Devices

(5.1) Using a computer

(5.2) E-mailing

(5.3) Printing documents

(5.4) Operating devices

(5.5) Operating the television remote control

(5.6) Using a mobile phone or smartphone

6. Leisure time

(6.1) Playing card and board games

7. Transport

(7.1) Driving a car

(7.2) Using a navigation system

(7.3) Using public transport

8. General

(8.1) Being responsible for his/her own medication

From the HIV questionnaire (7 items):

3. Administration

(3.9) Making online purchases (on any device)

6. Leisure time

(6.2) Playing computer games

(6.3) Reading a book or newspaper

(6.4) Organizing/initiating social activities

8. General

(8.2) Using keys

(8.3) Doing multiple things at once (multitasking)

(8.4) Writing in any format

From the neuro-oncology questionnaire (4 items):

4. Working

(4.2) Finishing work on time

6. Leisure time

(6.2) Following a TV program or movie

8. General

(8.5) Having a conversation with several people at the same time

(8.6) Learning new things (such as a course, computer program, or appliance)

Appendix B. Item evaluation by experts

Evaluation of the MS-IADL-Q went twofold:

Step 2.a. A first Dutch version of the questionnaire (Appendix B) was sent out to sixteen Dutch experts (12 females and 4 males). Experts were asked to anonymously rate the relevance of each item of the questionnaire on a visual analogue scale ranging from 0 ("not relevant at all") to 100 ("very relevant").[9] Additionally the clarity of the item description was evaluated and potential missing items could be added.

Step 2.b. After incorporating the suggested items by the Dutch experts, a second English version MS-IADL-Q (Appendix C) was sent out to twenty international experts (European and North-American; 12 females and 8 males) to investigate cross-cultural relevance of the included items and one more Dutch expert. The international experts needed to indicate relevance of each of the items in a similar fashion as was described in step 2.a. Independent samples *t*-test or Mann-Whitney U tests (in case of non-normal distributions) were used to assess cross-cultural validation. Statistical significance was set at $p < .05$. SPSS version 24 was used to perform statistical analyses. No significant differences were found between the Dutch and the non-Dutch expert ratings for any of the activities in the survey (data not shown).

In total, 15 national and 16 international experts filled in our survey. The response of one international expert was excluded as an outlier, since this respondent had an average Z-score of -2.96, and rated no activity higher than 30. The final expert group (N=30) consisted of 12 neurologists, six neuropsychologists, two clinical neuropsychologists, two scientists, three nurses, three rehabilitation physicians and two occupational therapists.

Appendix C. Item evaluation by patients and proxies

Participants were eligible for participation if they were 18 years or older. No exclusion criteria were included in the study. Patients and proxies were accessed by advertising via various online patient platforms (e.g., Facebook MS Centre Amsterdam, MS web, MS association Netherlands). Both patients and proxies received the Dutch version of the survey, in which they could also provide information about demographics and disease characteristics (MS type and disease duration).

Sixty-one PwMS (67% female, mean age = 49.0±10.2SD, relapsing remitting MS (N=37), progressive MS (N=17), "slowly progressive" (N=1), tumefactive MS (N=1) "RRMS, but most likely SPMS (although

not confirmed yet" (N=1), "not sure whether phenotype is RRMS or PPMS" (N=1), "I don't know" (N=3), and 30 proxies (57% female, mean age = 51.4±12.8SD) evaluated the 48 items of the MS-IADL-Q (Figure 1).

Appendix D. Second version of the MS-IADL-Q (after step 2.a; 48 items)

1. Household duties

(1.1) Carrying out household duties

(1.2) Grocery shopping independently

(1.3) Buying the correct groceries

(1.4) Cooking

(1.5) Preparing cold meals

(1.6) Making minor repairs to the house

2. Using household appliances

(2.1) Operating domestic appliances

(2.2) Operating the microwave oven

(2.3) Operating the coffee maker

(2.4) Operating the washing machine

3. Administration

(3.1) Paying bills

(3.2) Managing the household budget

(3.3) Using electronic banking

(3.4) Making appointments

(3.5) Keeping appointments (added by Dutch experts)

(3.6) Using a PIN code

(3.7) Obtaining money from an ATM

(3.8) Paying with cash

(3.9) Filling in forms

(3.10) Making online purchases (on any device)

4. Working

(4.1) Working

(4.2) Finishing work on time

(4.3) Focusing attention while performing tasks at work (added by Dutch experts)

(4.4) Dealing with distractions at work (added by Dutch experts)

5. Devices

(5.1) Using a computer

(5.2) E-mailing

(5.3) Printing documents

(5.4) Operating devices

(5.5) Operating the television remote control

(5.6) Using a mobile phone or smartphone

(5.7) Making phone calls with a mobile phone or smartphone (added by Dutch experts)

(5.8) Using social media on a smartphone (added by Dutch experts)

(5.9) Sending out e-mails on a smartphone (added by Dutch experts)

6. Leisure time

(6.1) Playing card and board games

(6.2) Playing computer games

(6.3) Following a TV program or movie

(6.4) Reading a book or newspaper

(6.5) Organizing/initiating social activities

7. Transport

(7.1) Driving a car

(7.2) Using a navigation system

(7.3) Using public transport

(7.4) Other participation in traffic (for instance by foot, bike, or scoot mobile; added by Dutch experts)

8. General

(8.1) Using keys

(8.2) Being responsible for his/her own medication

(8.3) Doing multiple things at the same time (multitasking)

(8.4) Having a conversation with multiple people at the same time

(8.5) Learning new things (such as a course, computer program, or appliance)

(8.6) Writing in any format

Appendix E.

Table 2. Evaluation of the MS-IADL-Q (version 2) by experts (national and international), People with MS (PwMS) and their proxies (ordered from highest average relevance to lowest average relevance). The average, median and interquartile range (IQR) of the relevance per group is displayed, together with the mean of all groups. For relevance, scores range from 0 (“not relevant at all”) to 100 (“very relevant”).

		**PwMS**			**Proxies**			**Experts**			**Mean of all**	
		Mean	Median	IQR	Mean	Median	IQR	Mean	Median	IQR	**Mean**	**Median**
Category	Activity											
4	3. Focusing attention while performing tasks at work	88.03	96.00	81.50-100.00	76.87	80.50	70.00-100.00	91.13	95.00	87.00-100.0	85.34	90.50
3	5. Keeping appointments	88.80	91.00	81.50-100.00	82.73	90.00	70.00-100.00	78.81	85.00	64.00-99.50	83.45	88.67
5	1. Using a computer	86.77	95.00	80.00-100.00	82.60	80.00	73.75-100.00	80.79	85.00	74.00-95.00	83.39	86.67
3	4. Making appointments	86.67	90.00	80.00-100.00	80.67	79.00	69.50-100.00	79.72	81.00	69.00-95.00	82.35	83.33
5	6. Using a mobile phone or smartphone	83.97	95.00	80.00-100.00	82.00	90.50	78.75-100.00	79.70	85.00	73.00-90.00	81.89	90.17

8	2. Being responsible for his/her own medication	88.34	99.00	81.00-100.00	81.07	90.00	70.00-100.00	75.77	81.00	58.75-97.75	81.73	90.00
8	5. Learning new things (such as a course, computer program, or appliance)	83.00	88.00	70.00-100.00	75.70	80.00	60.00-100.00	84.52	87.00	77.00-95.50	81.07	85.00
4	1. Working	81.51	90.00	69.00-100.00	70.80	80.50	50.00-100.00	89.21	90.00	85.00-99.50	80.51	86.83
8	4. Having a conversation with multiple people at the same time	81.03	81.00	70.00-100.00	77.57	80.00	60.00-100.00	82.57	85.00	75.00-95.00	80.39	82.00
3	6. Using a PIN-code	85.90	94.00	80.00-100.00	85.90	94.00	70.00-100.00	69.33	80.00	57.50-88.50	80.38	89.33
7	1. Driving a car	83.08	91.00	80.00-100.00	78.67	80.00	70.00-100.00	78.90	80.00	66.50-94.00	80.22	83.67
3	1. Paying bills	83.48	90.00	71.00-100.00	81.23	81.50	70.00-100.00	75.23	79.00	63.75-90.00	79.98	83.50
6	4. Reading a book or newspaper	82.26	89.00	72.00-100.00	78.90	80.00	60.00-100.00	78.40	80.00	69.75-90.25	79.85	83.00
4	4. Dealing with distractions at work	83.39	90.00	75.00-100.00	69.13	75.00	50.00-93.25	86.73	90.00	85.00-95.00	79.75	85.00
3	2. Managing the household budget	82.97	87.00	74.00-100.00	77.20	84.00	60.00-100.00	78.03	75.00	69.75-98.50	79.40	82.00

7	4. Other participation in traffic (for instance by foot, bike, or scoot mobile)	89.48	98.00	80.00-100.00	82.40	85.00	73.75-100.00	65.43	65.50	50.75-78.75	79.10	82.83
3	3. Using electronic banking	84.16	90.00	78.50-100.00	80.37	80.50	70.00-100.00	69.47	76.00	50.00-88.50	78.00	82.17
5	2. E-mailing	84.61	90.00	78.50-100.00	80.73	83.00	73.00-100.00	68.30	80.00	55.25-85.25	77.88	84.33
3	9. Filling in forms	84.54	90.00	78.50-100.00	78.43	80.00	67.75-100.00	69.46	75.50	60.00-84.25	77.48	81.83
1	4. Cooking	80.23	80.00	69.00-99.00	77.33	80.00	67.75-100.00	74.17	80.00	57.50-90.00	77.24	80.00
6	3. Following a TV program or movie	78.25	84.00	70.00-100.00	80.37	80.00	67.50-100.00	72.60	74.50	65.00-86.00	77.07	79.50
8	3. Doing multiple things at the same time (multitasking)	78.15	81.00	60.00-100.00	71.37	70.00	50.00-100.00	80.76	85.00	69.50-97.50	76.76	78.67
1	1. Carrying out household duties	79.44	80.00	70.00-96.00	75.80	80.00	60.00-100.00	74.48	75.00	67.00-90.00	76.57	78.33
5	7. Making phone calls with a mobile phone or smartphone	82.87	93.00	77.00-100.00	78.73	90.00	77.50-100.00	67.25	77.50	42.25-91.50	76.28	86.83
1	3. Buying the correct groceries	81.36	90.00	70.00-100.00	76.23	86.00	67.50-100.00	70.24	80.00	46.00-91.50	75.95	85.33

1	2. Grocery shopping independently	80.13	80.00	70.00-100.00	75.13	80.00	60.00-100.00	72.53	73.00	63.25-86.25	75.93	77.67
5	4. Operating devices	80.64	88.00	69.50-100.00	76.17	80.00	68.00-90.00	68.18	69.00	59.75-86.25	75.00	79.00
4	2. Finishing work on time	77.43	80.00	69.50-94.00	63.07	74.00	30.00-80.00	83.11	90.00	80.00-95.00	74.53	81.33
6	5. Organizing/initiating social activities	76.51	80.00	62.00-95.50	69.97	69.50	50.00-100.00	76.50	80.00	72.25-90.00	74.32	76.50
7	3. Using public transport	77.67	87.00	65.00-100.00	70.80	80.00	50.00-93.25	73.47	79.50	57.75-90.00	73.98	82.17
3	10. Making online purchases (on any device)	83.48	90.00	77.00-100.00	70.30	75.50	50.00-100.00	66.73	73.00	49.75-83.00	73.50	79.50
7	2. Using a navigation system	78.85	84.00	70.00-100.00	73.57	70.00	57.50-99.25	66.80	64.50	50.00-89.25	73.07	72.83
8	1. Using keys	79.02	88.00	70.00-100.00	77.13	80.50	58.00-100.00	58.82	62.50	34.25-90.00	71.66	77.00
8	6. Writing in any format	73.16	77.00	60.00-100.00	71.20	75.00	60.00-83.25	68.08	72.50	52.75-84.25	70.81	74.83
5	5. Operating the television remote control	76.67	88.00	60.00-99.50	75.83	80.00	64.00-100.00	58.23	62.50	39.50-82.50	70.25	76.83
2	2. Operating the microwave oven	72.95	80.00	53.00-97.50	77.13	80.00	63.75-100.00	59.53	64.50	35.00-81.75	69.87	74.83

2	1. Operating domestic appliances	76.18	80.00	66.00-99.50	73.67	80.00	64.00-90.00	59.19	65.50	44.75-75.00	69.68	75.17
1	5. Preparing cold meals	71.18	75.00	51.50-92.50	69.70	73.00	50.75-90.25	58.72	60.00	32.50-90.00	66.53	69.33
2	3. Operating the coffee maker	67.98	79.00	50.00-98.00	73.20	80.00	58.25-100.00	58.30	67.50	28.75-78.50	66.49	75.50
2	4. Operating the washing machine	72.07	80.00	58.00-99.00	63.43	72.50	39.75-92.50	60.03	66.50	46.25-81.50	65.18	73.00
3	7. Obtaining money from an ATM	67.46	71.00	50.00-99.00	60.40	60.00	28.75-100.00	66.60	73.00	51.25-85.75	64.82	68.00
5	9. Sending out e-mails on a smartphone	68.82	75.00	54.00-94.50	66.93	70.00	47.50-100.00	54.94	60.00	30.00-78.75	63.56	68.33
5	8. Using social media on a smartphone	69.02	72.00	51.00-98.00	67.27	73.50	50.00-100.00	53.44	56.00	31.00-70.00	63.24	67.17
5	3. Printing documents	62.23	62.00	49.50-80.00	65.57	70.00	47.50-90.50	55.40	61.50	30.75-80.00	61.07	64.50
3	8. Paying with cash	63.92	68.00	45.00-93.00	56.47	53.00	23.75-92.50	59.23	62.50	47.00-81.25	59.87	61.17
6	1. Playing card and board games	59.57	60.00	48.50-75.00	59.83	60.00	40.00-82.75	57.57	58.00	41.25-79.25	58.99	59.33
1	6. Making minor repairs to the house	64.67	69.00	50.00-85.00	62.07	60.50	39.75-83.50	44.72	50.00	24.50-59.50	57.15	59.83

6	2. Playing computer games	51.80	53.00	31.50-70.50	56.57	50.00	39.75-80.00	55.23	60.00	40.50-70.00	54.53	54.33

Appendix F. Amendment of items

Clarity | The two items “operating domestic appliances” and “operating devices” were considered to be vague and too broad. Therefore, the order of items was changed so that these activities would be the last in their respective categories (Appendix E). Addition of the word “other” (Appendix E, activity 2.4) allows for participants to answer the question the way they see fit to their personal circumstances. The item “finishing work on time” was rephrased to “completing your tasks at work on time”. Lastly, most of the comments on the two items “working” and “writing in any format” were considered to raise confusion as those items were considered to be mostly hampered by physical functioning”. This was solved by editing the format of the questionnaire: in the final version of the MS-IADL-Q, PwMS will first have to fill out whether items are applicable or not, and secondly whether its function is hampered by physical or cognitive problems.

Suggested items | Three items were suggested by all groups: “the ability to express your thoughts” “dealing with environmental stimuli” and “taking care of children”. The latter was merged with the suggestion “taking care of pets” to yield “taking care of others”. Additionally, “activities relating to self-care” was added to the questionnaire. One IADL that was mentioned remarkably often by PwMS was “planning daily activities” (n=11) and was therefore included in the questionnaire. We decided to split this item into two new items (i.e., “planning daily activities” and “carrying out this planning of daily activities”).

Finally, “dealing with environmental stimuli” and “doing puzzles” were listed as missed items by PwMS and proxies. Since two items were omitted from the category “leisure” (i.e., “playing card and board games” and “playing computer games”), the latter suggestion was combined with other hobbies such as sports and games to create a broader item: “undertaking hobbies and social activities as you wish to”.

Appendix G. Final version of the MS-IADL-Q (50 items)

1. Household duties

(1.1) Carrying out household duties

(1.2) Grocery shopping independently

(1.3) Buying the correct groceries

(1.4) Cooking

(1.5) Preparing cold meals

2. Using household appliances (change of order as suggested by PwMS/proxies)

(2.1) Operating the microwave oven

(2.2) Operating the coffee maker

(2.3) Operating the washing machine

(2.4) Operating other domestic appliances (rephrased)

3. Administration

(3.1) Paying bills

(3.2) Managing the household budget

(3.3) Using electronic banking

(3.4) Making appointments

(3.5) Keeping appointments (added by Dutch experts)

(3.6) Using a PIN code

(3.7) Obtaining money from an ATM

(3.8) Filling in forms

(3.9) Making online purchases (on any device)

4. Working

(4.1) Working

(4.2) Completing your tasks at work on time (rephrased)

(4.3) Focusing attention while performing tasks at work (added by Dutch experts)

(4.4) Dealing with distractions at work (added by Dutch experts)

5. Devices

(5.1) Using a computer

(5.2) Sending out e-mails (merged)

    (5.2.1) On a computer

    (5.2.2) On a smart phone (added by Dutch experts)

(5.3) Printing documents

(5.4) Operating the television remote control

(5.5) Using a mobile phone or smartphone

(5.6) Making phone calls with a mobile phone or smartphone (added by Dutch experts)

(5.7) Using social media on a smartphone (added by Dutch experts)

(5.8) Operating other devices

6. Leisure time

(6.1) Following a TV program or movie

(6.2) Reading a book or newspaper

(6.3) Undertaking leisure activities (added by PwMS/proxies)

(6.4) Initiating social activities

7. Transport

(7.1) Driving a car

(7.2) Using a navigation system

(7.3) Using public transport

(7.4) Other participation in traffic (for instance by foot, bike, or scoot mobile; added by Dutch experts)

8. General

(8.1) Using keys

(8.2) Planning daily activities (added by PwMS/proxies)

(8.3) Carrying out this planning of daily activities (added by PwMS/proxies)

(8.4) Taking care of self (added by PwMS/proxies)

(8.5) Taking care of others (children, pets, others) (added by PwMS/proxies)

(8.6) Being responsible for his/her own medication

(8.7) Doing multiple things at the same time (multitasking)

(8.8) Dealing with environmental stimuli (added by PwMS/proxies)

(8.9) Expressing your thoughts and feelings clearly (added by PwMS/proxies)

(8.10) Having a conversation with multiple people at the same time

(8.11) Learning new things (such as a course, computer program, or appliance)

(8.12) Writing in any format
